# *SuperCLEM*: an accessible correlative light and electron microscopy approach for investigation of neurons and glia *in vitro*

**DOI:** 10.1242/bio.042085

**Published:** 2019-05-20

**Authors:** Daniel G. Booth, Alison J. Beckett, Ian A. Prior, Dies Meijer

**Affiliations:** 1 Centre for Discovery Brain Sciences, University of Edinburgh, The Chancellor's Building, 49 Little France Crescent, Edinburgh EH16 4SB, UK; 2 Biomedical Electron Microscopy Unit, Department of Molecular and Cellular Biology, University of Liverpool, Crown Street, Liverpool L69 3BX, UK

**Keywords:** Myelin, SBF-SEM, Super-resolution light microscopy, 3D modelling, Sensory neuron, Node of Ranvier

## Abstract

The rapid evolution of super-resolution light microscopy has narrowed the gap between light and electron microscopy, allowing the imaging of molecules and cellular structures at high resolution within their normal cellular and tissue context. Multimodal imaging approaches such as correlative light electron microscopy (CLEM) combine these techniques to create a tool with unique imaging capacity. However, these approaches are typically reserved for specialists, and their application to the analysis of neural tissue is challenging. Here we present *SuperCLEM*, a relatively simple approach that combines super-resolution fluorescence light microscopy (FLM), 3D electron microscopy (3D-EM) and rendering into 3D models. We demonstrate our workflow using neuron-glia cultures from which we first acquire high-resolution fluorescent light images of myelinated axons. After resin embedding and re-identification of the region of interest, serially aligned EM sections are acquired and imaged using a serial block face scanning electron microscope (SBF-SEM). The FLM and 3D-EM datasets are then combined to render 3D models of the myelinated axons. Thus, the *SuperCLEM* imaging pipeline is a useful new tool for researchers pursuing similar questions in neuronal and other complex tissue culture systems.

## Abstract (precis)

**Summary:** We developed an advanced, accessible and adaptable correlative light and electron microscopy pipeline for study of neuron-glia cultures and demonstrate its power by deriving accurate 3D models of myelinated axons.

## INTRODUCTION

Increasingly more sophisticated and powerful light microscopic (LM) techniques are being used to acquire spatial, temporal and functional information on proteins in cells and tissues (Fornasiero and Opazo, 2015). The combination of this LM information with ultrastructural information, acquired through electron-microscopic (EM) techniques, has revolutionised biology and deepened our understanding of how ‘form follows function’ (Sullivan, 1896). Such correlative light and electron microscopic (CLEM) approaches reveal the fine details of proteins in mitochondria, mitotic chromosomes, neuronal synapses and glial cells in the nervous system of a range of organisms (Begemann and Galic, 2016; Booth et al., 2016; Burel et al., 2018; de Boer et al., 2015; Kopek et al., 2017; Lees et al., 2017; Smith and Starborg, 2018). For example, a recent study used array-tomography, wide-field fluorescence microscopy and transmission electron microscopy (AT-TEM) to demonstrate that different classes of interneurons synapse onto specific dendritic domains of hippocampal CA1 pyramidal neurons and that this specific architecture of inhibitory connectivity contributes to dendritic computation (Bloss et al., 2016). However, these highly sophisticated CLEM techniques require specialist equipment, training and complicated software and are therefore largely reserved for specialists (Peddie and Collinson, 2014).

We sought to develop an accessible CLEM approach to study the interaction between neurons and glial cells as they shape the function and structure of the nervous system. Currently, complex cellular interactions between these cells can be studied *in vitro* in controlled culture systems. Of particular relevance here are co-cultures of dorsal root ganglia (DRG)-derived primary sensory neurons and glia co-cultures in which myelination of axons is achieved by Schwann cells or oligodendrocytes (Kleitman et al., 2002; Taveggia and Bolino, 2018). An advantage of this system lies in the fact that cultures can be easily established from DRGs and glial cells of genetically modified (mutant or fluorescent reporter) animals or through Crispr/Cas directed modifications of normal cells to allow tracking of proteins of interest over extended periods of time. These specific attributes make this culture system a useful approach to study developmental aspects of myelination and to model myelinopathies and axonopathies (Kegel et al., 2014; Kleitman et al., 2002; Melli and Höke, 2009; Reilly and Shy, 2009; Robinson et al., 2018; Spiegel et al., 2007; Taveggia et al., 2005; Trimarco, 2014). However, a highly desirable goal in such studies is to correlate functional aspects of cell interactions, as revealed by LM, with volumetric ultrastructural information that can only be obtained through EM.

Here we describe the development of a simple imaging pipeline in which we combine super-resolution LM and serial block-face scanning EM (SBF-SEM) to digitally create three-dimensional (3D) models of myelinated axons and nodes of Ranvier, using off-the-shelf Amira™ imaging software. A major obstacle in all CLEM approaches is to reliably identify fiducials to correlate LM with EM images, especially in dense cultures or tissues. We overcome this problem by combining a tiling strategy with biological fiducials, which allows the reliable correlation of images without destructive marking. Thus, our straightforward imaging pipeline provides an accessible and adaptable approach to study neuronal and other complex culture systems and tissues.

## RESULTS

### Preparation of samples for *SuperCLEM*

**Fig. 1.**
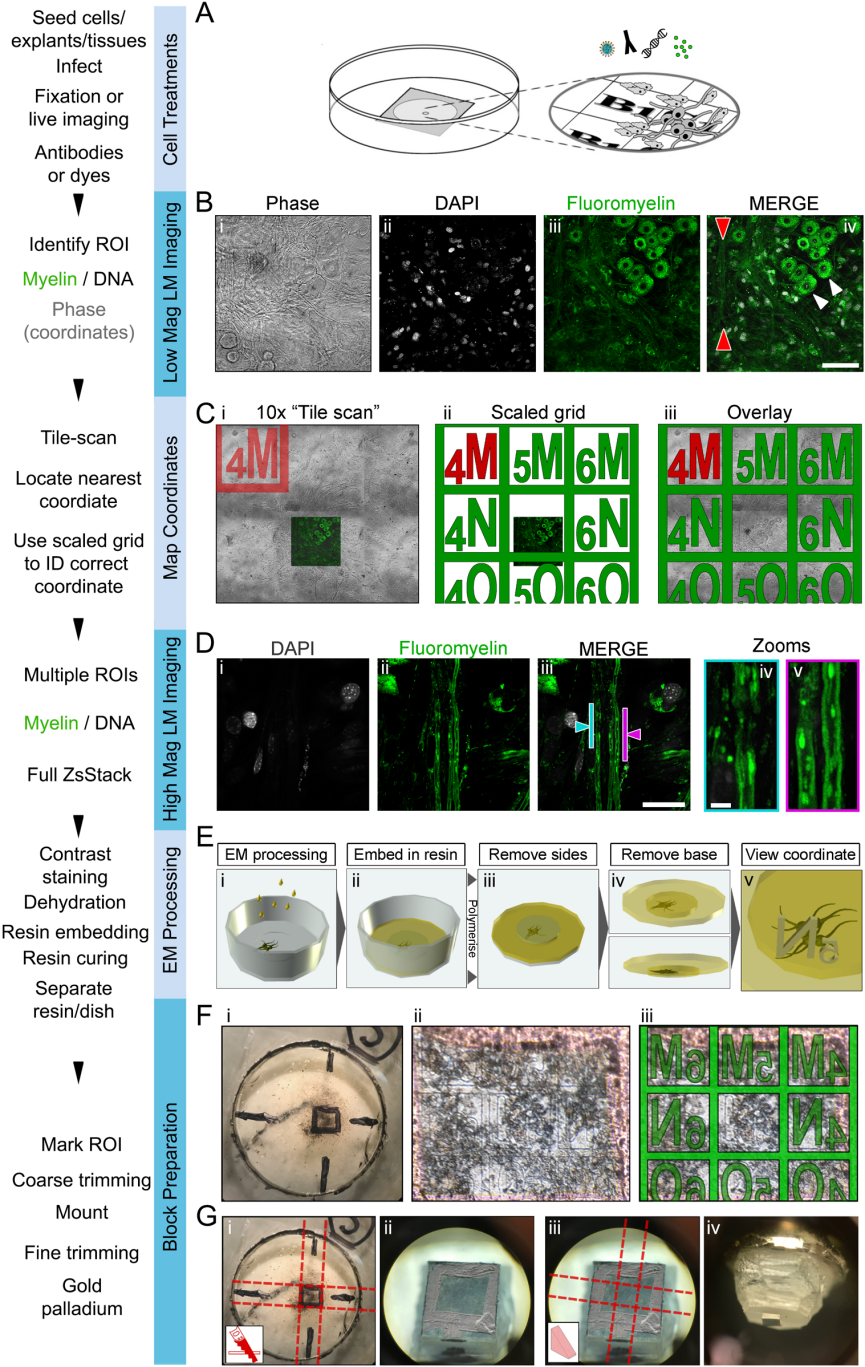
**Selection of ROI and LM imaging.** (A) Schematic showing DRGs seeded onto a gridded dish (MatTek). (B–D) Acquisition of optical images. (B) Overview images (20× objective) acquired to identify a ROI containing myelinated axons (red arrowheads). Panels show: (i) phase contrast, (ii) DAPI stain, (iii) Fluoromyelin Green staining and (iv) merge. White arrowheads indicate neuronal soma. (Ci) A 5×5 tile scan was performed using a 10× objective to acquire phase contrast images. Neuronal explants and neurite outgrowth masked the coordinates usually visible directly below, however the coordinate ‘4M’ close to the ROI was visible. (Cii) A scaled map of the grid was generated in Photoshop (Adobe) using the nearest visible coordinate ‘4M’ (red) as a fixed source for location of other predicated coordinate positions (green). (Ciii) The grid was overlaid with the 20× image acquired previously (Bi), confirming that the ROIs all reside in ‘5N’. (D) High magnification (100×) super-resolution images of the ROI in A. Panels show (i) DAPI stain, (ii) myelin and a (iii) merge of the two. Arrows point to two ROIs; (blue) a node of Ranvier and (pink) an extended region of myelinated axon, magnified in iv and v, respectively. (E) A workflow demonstrating (i) EM processing, (ii) embedding the samples in resin, (iii) removal of the plastic sides of the dish with side-cutting pliers and (iv) separation of the base from the resin. Once separated, the previously identified coordinate was visible, imprinted on the resin (v). (F) The area containing the coordinate was marked (i). Although partially masked by the sample the coordinate was still visible (ii) and confirmed using an overlay of the grid (iii). (G) Coarse resin removal by (i) four cuts with a junior hacksaw, leaving a block face (ii) roughly the size of the region marked earlier. The coarse block was glued onto a cryo-pin (iii) and fine trimming (iv) was performed using an ultra-microtome. Scale bars: (B) 100 µm, (Diii) 30 µm, (Div) 5 µm.

DRG tissues were dissected from E13 embryos or postnatal day (P) 4–6 pups of wild-type mice, seeded onto gridded dishes (MatTek) (Fig. 1A) and cultured as explants for 7 days, according to established protocols (Kegel et al., 2014; Kleitman et al., 2002; Sleigh et al., 2016; Taveggia and Bolino, 2018), to allow neurite outgrowth and expansion of the endogenous Schwann cell population. Myelination was induced through addition of ascorbic acid to the culture medium and after an additional 14 days of *in vitro* cultures, myelinated samples were processed for *SuperCLEM*. The choice of methods used to label desired proteins/organelles was governed by availability and/or amenability to the desired cell-types/tissues and in this case, we used cell permeable dyes to fluorescently label nuclei (DAPI) and myelin (Fluoromyelin Green).

The sample was transferred to a Zeiss LSM 880 Confocal Microscope with Airyscan, and overview images (magnification 20×) were acquired to identify regions of the dish containing myelinated axons, as identified by DAPI and Fluoromyelin Green staining (Fig. 1Bi–iv). A region of interest (ROI) was selected (Fig. 1Biv, red arrowheads). Imaging at high magnification (Fig. 1D; 100× magnification) allowed the identification of two desired structures; (1) a node of Ranvier (identified via a gap in myelin) and (2) an extended stretch of myelinated axon (blue and pink bars, respectively, Fig. 1Diii). These structures are the focus of our ultra-structural analysis by *SuperCLEM.* Extended Z-stacks were acquired that not only contained lateral information of the structures of interest, but also other flanking structures, such as cell soma (Fig. 1Biv, white arrow). These large and morphologically distinct structures act as biological fiducials (Fig. S1A).

### Using tile-scans to identify ‘finder’ coordinates

A major obstacle when using CLEM for the analyses of complex and expansive multi-cellular cultures such as myelinating co-cultures is revisiting the cells or structures of interest by EM. Dense layers of cell bodies and neurites in the co-cultures (Fig. 1Bi) mask the relocation coordinates. To overcome this for DRG cultures we developed a simple strategy using tile scans (Fig. 1C). The extended field of view allowed the identification of the nearest visible ‘finder’ coordinate (Fig. 1Ci; Fig. S2A,B). Overlaying the tile scan with a scaled image of the grid (Fig. 1Cii) allowed the masked ‘finder’ coordinate to be identified (Fig. 1Ciii).

The sample was next processed for SBF-SEM (Fig. 1Ei), embedded in resin, cured (Fig. 1Eii) and excised from the dish (Fig. 1Eiii–iv), as previously described (Booth et al., 2013). Relocation coordinates were visible embossed on the underside of the resin (Fig. 1Ev). Using a dissection microscope an area corresponding to approximately nine coordinates (with the coordinate of interest in the centre) was marked (Fig. 1Fi). This was possible as the finder coordinates were still visible (Fig. 1Fii), including the region of interest (Fig. 1Fiii) that was masked when viewed with transmitted light (Fig. 1Bi). To remove as much excess resin as possible, the block face was coarse-trimmed using a junior hacksaw (Fig. 1Gi–ii), glued onto an aluminium pin and then precision-trimmed using an ultra-microtome (Leica) (Fig. 1Giii,iv; Fig. S2C). The sample was coated with gold palladium (AuPd) before mounting into the Gatan 3View unit inside a FEG250 Quanta ESEM (FEI) in preparation for imaging.

### SBF-SEM imaging and correlation of optical and EM datasets

**Fig. 2.**
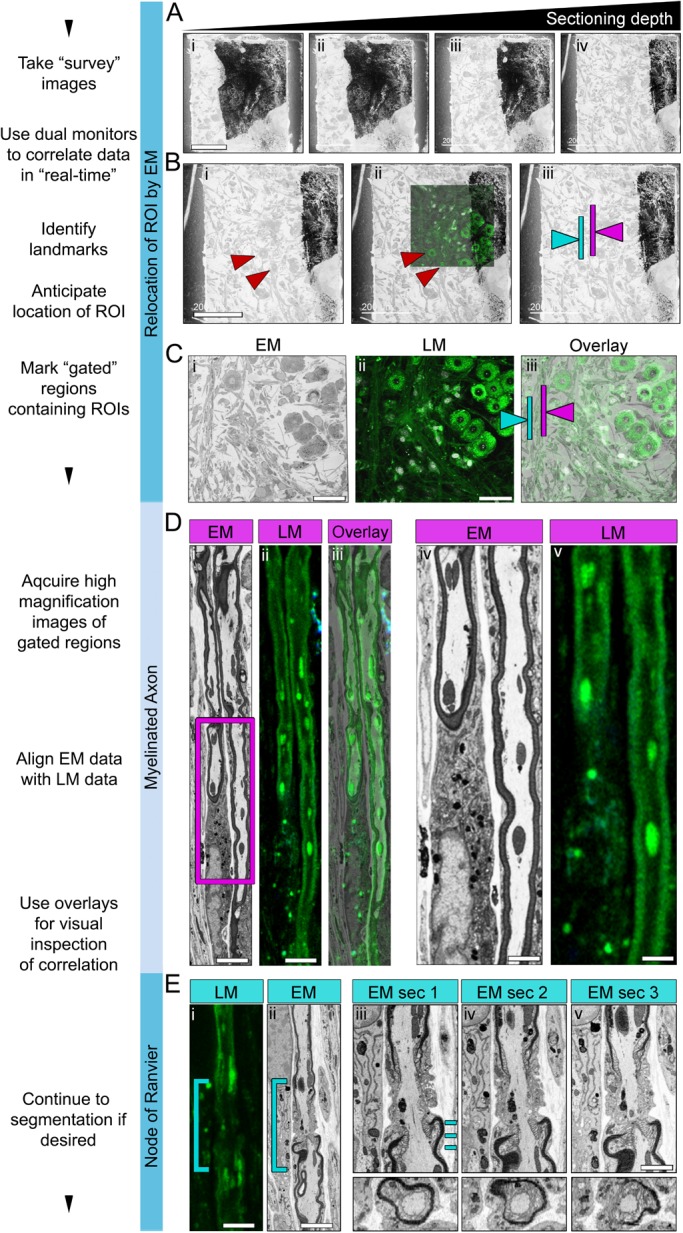
**Correlating LM and EM data.** (A) Four progressive ‘survey’ sections (i–iv) taken at depths of approximately 5, 10, 15 and 20 µm. (B) The first ‘survey’ EM orthoslice (at a depth of ∼20 µm) showing obvious landmarks (i), such as cell soma (red arrows), to be correlated with fluorescent optical sections (ii) and areas of the block anticipated to contain ROIs (iii, pink and blue arrows). (C) Overview images of the first physical (i) and optical (ii) sections that have the correlating ROIs (iii, pink and blue arrows, showing myelinated axons and a nodal region, respectively). (D) High-resolution correlative imaging of an extended region of myelinated axon. Panels show high-resolution EM (i,iv) and LM (ii,v) images, and an overlay of LM and EM images (iii). LM voxel size, 0.068×0.068×0.3 µm (acquired using a 100× objective). EM voxel size, 13.8×13.8×80 nm (acquired at 1972×). (E) High-resolution correlative imaging of node of Ranvier, showing high-resolution (i) LM and (ii) EM data, and (iii–v) high-magnification examples of consecutive EM sections viewed either longitudinally (upper panels) or orthogonally (lower panels). Blue dashes in Eiii mark the level at which the orthogonal sections were made, shown in Eiii–v, lower panels. Scale bars: (A,B) 200 µm, (C) 100 µm, (Di,ii) 5 µm, (Div,v) 3 µm, (Ei,ii) 5 µm and (Ev) 3 µm.

To maximise the chances of successful correlation (i.e. revisiting the same structure in both LM and SBF-SEM), we used a dual monitor workstation that allows both the optical sections and ‘live’ acquisition of EM data to be viewed simultaneously (Fig. S3A). Consecutive survey sections (∼200 nm thick) were acquired (Fig. 2A) until biological fiducials were revealed, present in both the LM and EM datasets (Fig. 2Bi,ii, red arrows). These ‘landmarks’ allowed the position of yet-to-be-sectioned targets to be accurately estimated, in both X-Y (Fig. 2Biii) and Z planes (Fig. S1). At this stage the structures of interest were ‘gated’, meaning the field of view was decreased and the magnification increased. The section thickness was now also decreased (80 nm), to improve the Z-resolution (Fig. S1B). In total 300 images at 80 nm intervals were acquired.

Next, the LM and SBF-SEM stacks were visually inspected for successful correlation (Fig. 2D,E). Representative images of two gated ROIs; an extended region of myelinating axon (Fig. 2Di–v) and a node of Ranvier (Fig. 2Ei–v), are shown. Overlays of the super resolution optical sections and SBF-SEM sections confirm that the *SuperCLEM* imaging pipeline was successful (Fig. 2D).

To further improve the degree of correlation between imaging modes, which is rarely perfect (compare Fig. S3B with S3D–F), both data-sets were digitally re-sectioned across all angles using the Amira™ Multiplanar Tool (Fig. S3C), thus maximising the alignment (Fig. S3F).

To estimate the accuracy of correlation we performed a bespoke shift-analysis. To limit the impact of differences between optical and physical section thickness, projections were generated from sections that contained axons (Fig. S4A). The optical stack projection consisted of 9×300 nm sections (2.7 µm in total). The EM projection consisted of 33×80 nm sections (2.64 µm in total). A grid of concentric rings (ImageJ ‘concentric circles’, 6.5 µm spacing) was placed over both projections (Fig. S4B), centred on a clear landmark observable in both projections. Next the position of structures/objects clearly identifiable in both projections (myelin–Fluoromyelin Green, nuclei–DAPI) were marked. The resulting coordinates were mapped onto a 2D-scatter plot (Fig. S4Biii) and the shift measured for each point. The mean shift was 0.24 µm (±0.17 µm). No obvious relationship was observed between the point-shift and either the distance from the origin or its overall position (Fig. S4C), suggesting that any shrinkage artefacts were not directionally uniform.

### Segmentation of SBF-SEM data to generate 3D models

**Fig. 3.**
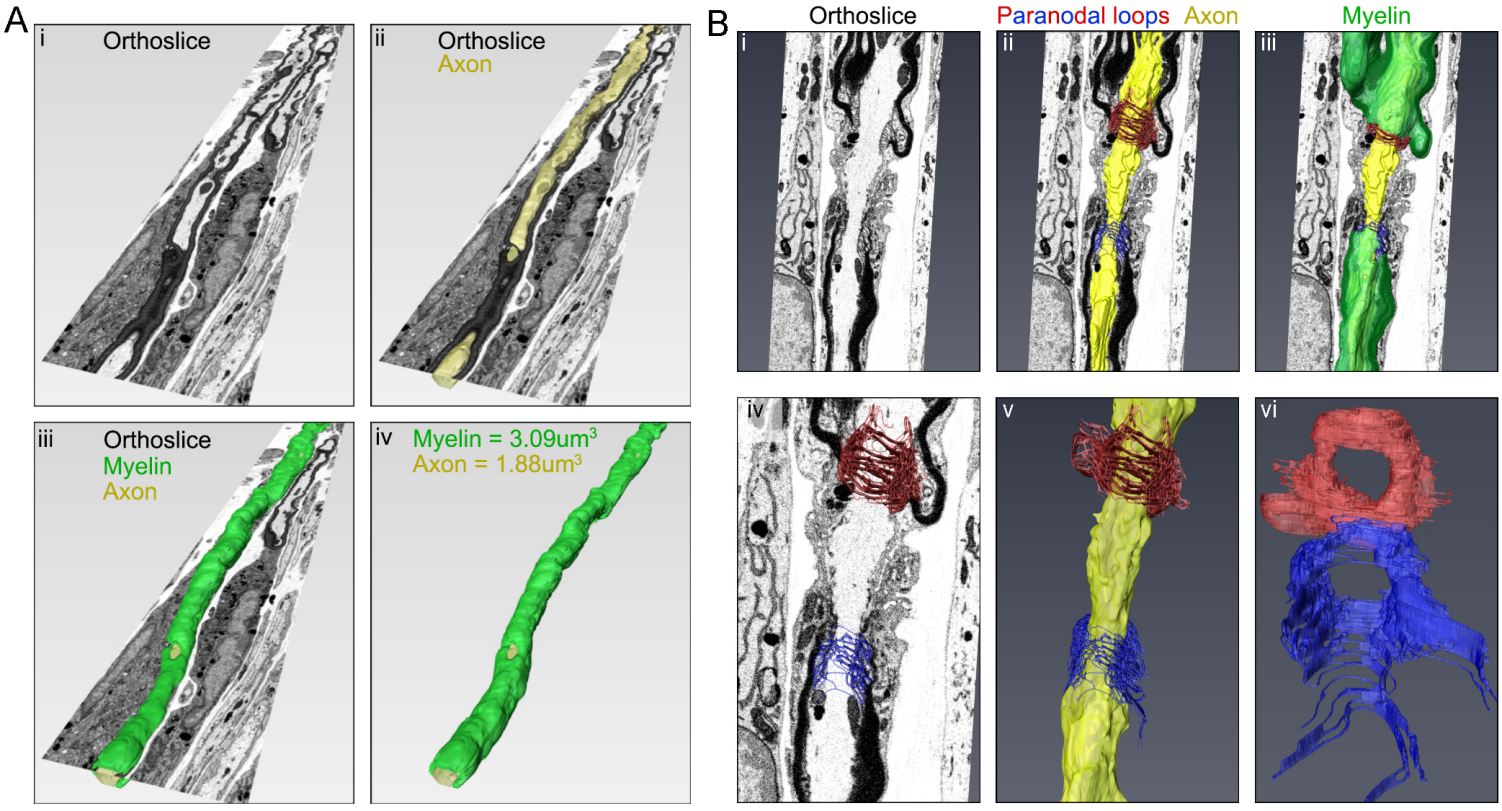
**Render of a myelinated axon and node of Ranvier.** (A) Representative images of one of the four axons reconstructed from ∼300 consecutive 3D-EM sections using Amira™. (i) Representative EM orthoslice and cross-section of a myelinated axon. (ii) EM orthoslice traversing a reconstructed axon (yellow). (iii) EM orthoslice traversing a reconstructed fibre, consisting of the axon (yellow) and myelin (green). (iv) Digitally reconstructed fibre consisting of axon (yellow) and myelin (green). Volume measurements are indicated. (B) Digital image synthesis of a node of Ranvier from 66 consecutive EM sections. (i) Representative orthoslice. (ii) Render of the axon (yellow) and paranodal loops (red and blue). (iii) Render of axon (yellow), myelin (green) and paranodal loops (red and blue). (iv–vi) Higher-resolution zooms of paranodal loops traversing the orthoslice (iv), with the axon (yellow, v) or alone (vi).

A further strength of the *SuperCLEM* imaging pipeline is the ability to generate nanometer accurate 3D models or renders from the SBF-SEM data. Surface volumes were created by segmenting structures of interest in the SBF-SEM datasets using the ‘blow tool’ (Fig. S3Gi) across multiple layers (Fig. S3Gii–iv). Three extended regions of myelinated axon, with a combined length of ∼400 µm were modelled (see also Movie 1). This included surface volumes of both the axon (Fig. 3Aii) and myelin (Fig. 3Aiii), allowing a variety of useful parameters to be retrieved, including compartment volumes (Fig. 3Aiv). Next, a surface renders of a node of Ranvier, including the axon (Fig. 3Bii), myelin (Fig. 3Biii) and paranodal loops (Fig. 3Biv,vi) was generated. The resolution of the model allowed us to detect that both clusters of paranodal loops (red and blue renditions in Fig. 3B, from either side of the node) each was comprised of 10–11 lamellae. This number of lamellae is expected for a small diameter axon (1–2 µm) and importantly corresponds to empirically derived numbers (Friede and Samorajski, 1967).

### Using *SuperCLEM* to study morphometrics

Next, we exploited the LM and SBF-SEM datasets to retrieve and compare a variety of diagnostic geometric parameters which allow for the g-ratio to be calculated (Fig. S5A) including myelin diameter (Fig. S5B) and axon diameter (Fig. S5C). The g-ratio (=d/D) relates the inner diameter of the myelinated fibre (d=axon diameter) with the outer diameter (D=axon+myelin diameter) and has a relatively constant value of ∼0.6, as observations have revealed that myelin thickness grades with axonal diameter (Donaldson and Hoke, 1905).

**Fig. 4.**
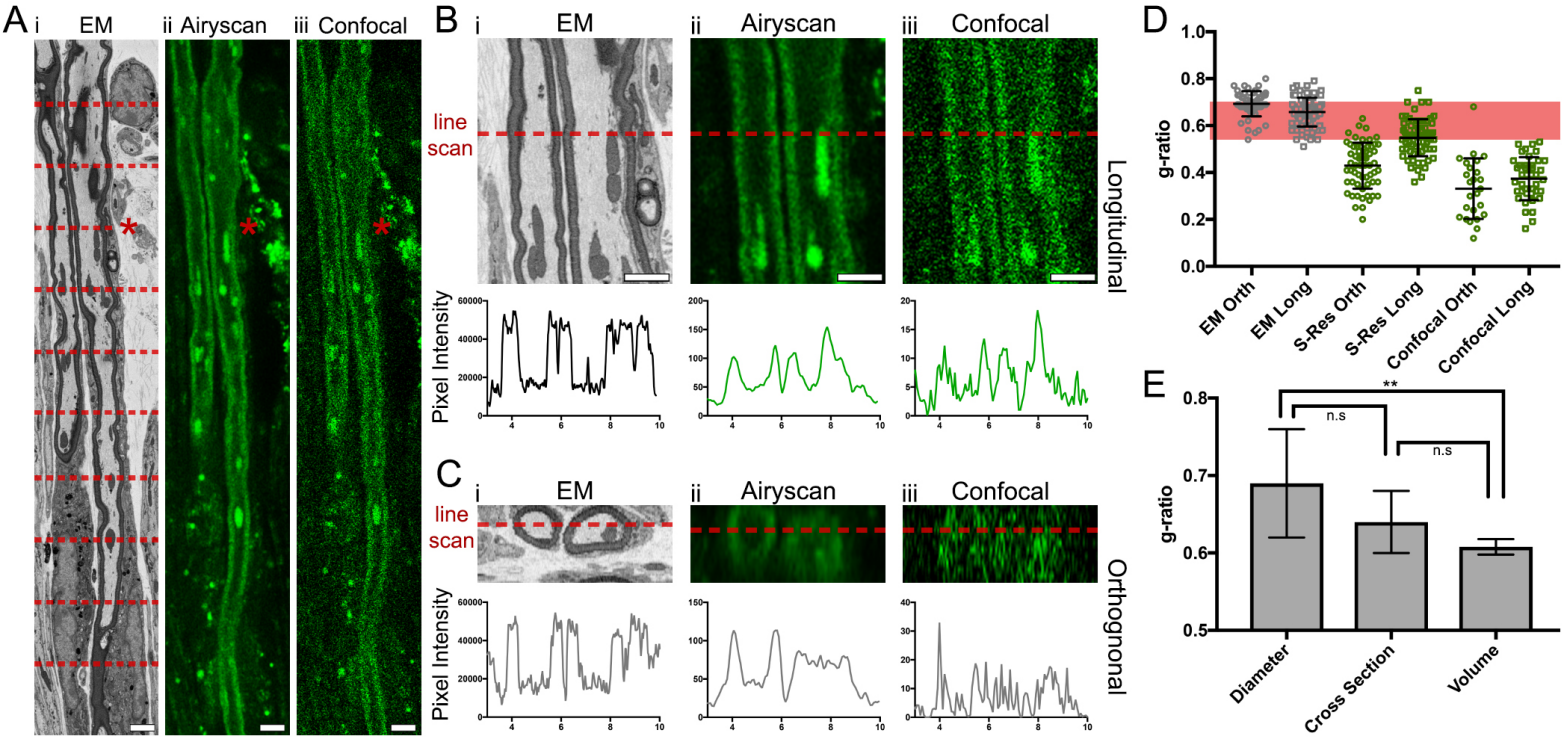
**Using *SuperCLEM* to retrieve and compare morphometric data.** (A­–C) Representative overview images used to perform pixel density scans (red dashed lines) of identical regions of an axon imaged by (i) EM, (ii) Zeiss Airyscan and (iii) confocal microscopy. The asterisks in A indicate the position of the representative line-scans shown in B and C. Pixel density scans were acquired from identical regions in both B(i–iii) longitudinal and C(i–iii) orthogonal orientations. Line scans were used to measure axon diameter (d) and fibre diameter (D) to calculate the g-ratio. (D) Graph comparing g-ratios estimated from data represented in A–C, calculated using diameter measurements. Each point represents one g-ratio measurement. *N*=3 axons, *N*=100 line scans. Note that due to excessive ‘noise’ created by out-of-focus light, some measurements could not be obtained from matching regions found in confocal images (*N*=3 axons, *N*=45 line scans). (E) A bar graph comparing mean g-ratio measurements acquired using diameter, cross sectional area or volume (from 3D modelling data). Bars denote standard error of the mean. Statistical significance between datasets was registered using an analysis of variance (ANOVA). n.s., not significant, **P<0.01. Scale bars: (A) 4 µm and (B) 2 µm.

We acquired line scans at set intervals along the length of the imaged axon from both longitudinal (Fig. 4A,B) and digitally re-sectioned orthogonal SBF-SEM datasets (Fig. 4C). The resulting traces revealed inner diameter (d) and outer diameter (D) used for calculating the g-ratio (See Fig. S5 for raw diameter data). Mean g-ratios of 0.66±0.06 and 0.69±0.05 were found for the longitudinal and orthogonal EM data respectively (Fig. 4D). Same area analysis in data acquired using the Airyscan (super resolution) retrieved mean g-ratios of 0.43±0.1 and 0.55±0.08 for orthogonal and longitudinal sections respectively (Fig. 4D). Mean g-ratios of 0.37±0.1 and 0.33±0.13 were calculated for orthogonal and longitudinal sections obtained in confocal mode (Fig. 4D). Thus, our data showing that g-ratios derived from most LM data deviate significantly from the empirical values as well as the theoretical value (Fig. 4D, red bar) clearly indicate the poor diagnostic value of LM in accessing structural aspects of myelin in culture. We next revisited our SBF-SEM data to compare g-ratios measured using alternative parameters. We calculated and compared g-ratios using diameter (d axon/D fibre), cross-sectional area (ca axon/CA fibre), and volume from three myelinated fibres. The mean g-ratios from diameter (0.69±0.07), cross-sectional area (0.64±0.04) and volume (0.61±0.01) were directly compared (Fig. 4E). Thus, whereas all three methods yield g-ratios that fall within the empirical range, the volume-based method produces the most accurate value (lowest standard deviation) that corresponds exactly with theoretical predictions.

## DISCUSSION

Here we have designed and tested an advanced yet accessible 3D light and electron microscopy pipeline to correlate functional and structural aspects of myelinating neuron-glia cultures. We describe the procedure in great detail, starting from myelinated cultures to 3D modelling of myelinated axons, including the node of Ranvier and the paranodal myelin loops and demonstrate how these models can be used to derive accurate g-ratios from a small number of modelled axons.

### Microscopy improvements leading to *SuperCLEM*

Volumetric EM datasets can be obtained through a variety of techniques involving transmission or scanning EM. Whereas serial-section TEM in principle offers higher resolution, it is technically more demanding than the available scanning blockface techniques FIB-SEM and SBF-SEM (see Kremer et al., 2015 for an historic oversight and in-depth discussion of the pros and cons of the different techniques). Since its introduction by Denk and Horstmann (2004), SBF-SEM has been widely used to obtain nanometer scale renderings of large multi-cellular structures/tissues (Peddie and Collinson, 2014). The application of both FIB-SEM and SBF-SEM have had a particularly large impact in the neurosciences, a development that was aided by the introduction of improved staining procedures (Ohno et al., 2014; Tapia et al., 2012).

These improvements in 3D-EM have somewhat mirrored advances with light microscopy, such as the super-resolution imaging of axons (Dani and Huang, 2010; Lakadamyali et al., 2012; Sigal et al., 2015) and have created an opportunity for the development of new multi-modal imaging strategies in the form of super resolution correlative light electron microscopy (SR-CLEM) (Kopek et al., 2017). However, these tools are still very much in their infancy and the availability of accessible protocols is limited. Hence, to further our research we developed an accessible imaging pipeline that is designed around the super resolution provided by the Airyscan confocal microscope and the acquisition of EM data through SBF-SEM.

### Correlating LM and EM datasets

A critical step in the correlation of LM and EM data is the re-identification of structures of interest. Near-infrared branding is one of the approaches used to overcome this problem (Bishop et al., 2011; Lees et al., 2017) as it allows permanent marks or fiducials to be etched in the sample close to the region of interest. However, this method requires specialised equipment (laser) and is destructive. Polymer beads can also be used (Kukulski et al., 2012), however this also results in obscuring regions of interest. A more desirable approach is to use relocation coordinates etched into the base of gridded dishes. However, a common challenge surfaces when analysing structures within dense beds of multi-cellular cultures/tissues, which masks the coordinates, essentially camouflaging the position of targets in both X-Y and Z planes. This issue may be avoided when analysing simple cultures consisting of monolayers of well-dispersed cells (Booth et al., 2013).

Our *SuperCLEM* workflow incorporates a two-step system to overcome this technical obstacle. (1) To revisit the X-Y position of the target we used tile scans to identify the next nearest visible coordinate. (2) To revisit the Z-position of the target we used biological fiducials. During LM imaging we acquired comprehensive Z-profiles, i.e. acquired a Z-stack which also contains several optical sections transversally flanking the sections that contain any structures of interest (Fig. S1A). This allowed useful ‘landmarks’ to be identified in these flanking sections. For example, in the overview images (Fig. 1Aiv), numerous cell somas can be seen. These are large structures that appear in both the earlier and later optical sections, i.e. flanking the structures of interest (Fig. S1A,B) and act as a reference point in both X-Y and Z planes. This system also makes the SBF-SEM more time efficient as it removes the need to acquire ultrathin (80 nm) sections throughout the whole stack. Instead, thicker survey sections can be taken until the position of the structure of interest is reached. The sectioning depth to reach the target is sample-dependant and in our case, although some axons run close to and parallel with the base of the dish, many others traverse the uneven surface of bedded fibroblasts and glia.

### Accuracy of correlation between LM and SBF-SEM data

It is important to note that the degree of correlation between LM and EM datasets is rarely perfect, using any format of CLEM. This is likely to be a compound effect of numerous factors, including; (1) processing artefacts, (2) differences in the thickness of optical and physical sections and (3) disparities in how the sample is seated, with reference to imaging using a light or electron microscope. The latter can be influenced by the sample to pin-mounting angle and also the approach angle of the diamond knife. We demonstrated that LM to EM registration can be improved by digital re-sectioning of the datasets and using projections to account for section thickness. Any remaining differences are likely due to processing artefacts. Our system also allowed the presence of anisotropic shrinkage to be determined by measuring the distance from origin (the centre of the scatter plot) and the angle of each point relative to the centre line. Indeed, we found a mean shift of 240 nm between LM and SBF-SEM data. To put this into context, this shift is less than half the thickness of the myelin, suggesting a good degree of correlation.

### *SuperCLEM* and the g-ratio

The near constancy of the ratio of axon diameter (d) over myelinated fibre diameter (D) was first reported by Donaldson and Hoke (1905) and later confirmed by many others, including Gasser and Grundfest (1939) who established that the speed of action potential propagation correlates with fibre size (D). Theoretical and computational approaches determined that conduction speed and fidelity is maximized around d/D (=g-ratio) values of 0.60–0.62, dropping off at higher (hypomyelinated axons) and lower (hypermyelinated axons) values (Deutsch, 1969; Rushton, 1951). Empirical values for a wide range of fibre diameters in healthy peripheral nerves (Chomiak and Hu, 2009) correspond well to these theoretical values. Thus, as the g-ratio is a relatively sensitive measure for nerve health and maturity, we examined how well the g-ratio of myelinated fibres in our cultures correspond to this theoretical optimum as a means of assessing the structural maturity of the myelinated axons.

These values, retrieved from the SBF-SEM datasets, fall within the empirical range of g-ratios (0.55<d/D>0.68) reported for normal peripheral nerve fibres of the sciatic nerve (Chomiak and Hu, 2009), thus suggesting that the myelinated axons in our cultures are structurally matured. There is a general acceptance that g-ratios can be derived reliably only from EM images and not from LM images (Dyck and Thomas, 2005). It is surprising that this has never been stringently tested. We exploited our imaging pipeline to directly compare g-ratios derived from different modes of microscopy to those obtained from EM analysis and as might be expected most measurements acquired fell below the empirical range. However longitudinal g-ratio measurements acquired using the Airyscan were just within the empirical range, suggesting that this mode of imaging may have sufficient resolution to attain accurate g-ratios. Due to this borderline result, we recommend using such data with caution or with support from other qualified imaging tools or staining methods.

The most commonly used measurements for calculating g-ratios derive from the diameters or cross-sectional areas of the axon and myelinated fibre in EM images of transverse sections of nerve. An obvious drawback is that axonal fibres in such preparations usually deviate significantly from a perfect cylinder along the fibre axis, and some fibres will be sectioned at an oblique angle (Friede and Samorajski, 1967). To overcome this issue, the calculation of g-ratios from volumetric data will average out local structural deviations along the fibre axis. While no significant differences in g-ratios were found between diameter and cross-sectional measurements, a significant difference was observed between diameter and volumetric measurements with volumetric data providing tighter values with small standard deviation. Additionally, the volume-based g-ratio value corresponds exactly to the value predicted by theory [0.6–0.62; (Deutsch, 1969; Rushton, 1951)] further reinforcing the suggestion that indeed these myelin fibres are structurally mature.

As for the usefulness and versatility of *SuperCLEM* for other applications, we have been able to identify and image the axon initial segment (AIS) (Fig. S6A–E, Movie 2) of a sensory neuron. These images showed the typical arrangement of satellite glial cells flanking the AIS (arrows). Other suitable and actively studied organelles could include mitochondria. Dyes such as MitoTracker (Thermo Fisher Scientific) are well characterized and could be readily incorporated into the *SuperCLEM* pipeline. Further developments of this imaging pipeline will include the incorporation of STED super-resolution microscopy to further narrow the resolution gap between light and electron microscopic datasets.

In conclusion, we have developed here a novel multi-modal microscopy tool (*SuperCLEM*) and validated its utility in measurements of myelin maturation in DRG cultures, the most prominent *in vitro* system for modelling myelinating diseases. We addressed fundamental issues with classical g-ratio measurement approaches by use of 3D morphometry and expanded the robustness of *SuperCLEM* to identify and model discrete neuronal structures, including a node of Ranvier. In addition to its value in the study of myelinating neuron-glia cultures, our approach should be applicable to a wide range of complex culture systems, in particular organoid cultures in which complex developmental processes can be modelled.

## MATERIALS AND METHODS

All animal work was performed in compliance with UK legislation (Scientific Procedures Act 1986) and was approved by the University of Edinburgh Ethical Review Board.

### Isolation of DRG neurons and light microscopy imaging

DRGs were dissected from C57Bl6 mouse embryos at embryonic day 13 or P4–6 pups (sex not determined) according to established protocols (Kegel et al., 2014; Kleitman et al., 2002; Sleigh et al., 2016), seeded onto gridded dishes (MetTek) and maintained in MEM (Gibco) supplemented with 3% fetal bovine serum (FBS), 50 ng/ml NGF and penicillin and streptomycin in a 5% CO_2_ incubator at 37˚C. After 7 days *in vitro* (DIV7), myelination was induced by addition of ascorbic acid to the medium (final concentration 50 µg/ml) (Kegel et al., 2014; Taveggia and Bolino, 2018). The cultures were maintained for another 14 days to allow myelination to proceed. Once myelinated, samples were prepared for *SuperCLEM*, starting with optical imaging. Cultures were fixed in warm fixative (2% glutaraldehyde, 2% paraformaldehyde in 0.1 M sodium cacodylate buffer) for 1 h. Following three washes with PBS, cells were labelled with 4′,6-diamidino-2-phenylindole (DAPI) (Thermo Fisher Scientific) and Fluoromyelin Green (Thermo Fisher Scientific), both 1 in 300 dilution, in PBS for 30 min. Following 3×5 min washes, samples were transferred to an LSM880 Zeiss Airyscan microscope and imaged using a 20× air objective to identify cells and structures of interest. Next, a ‘tile-scan’ was acquired using phase contrast. The ROI was located and the coordinate(s) and flanking coordinates for the ROI identified. Z-stacks of the ROI were acquired using an alpha Plan-Apochromat 100×/1.46 oil objective. Images were acquired using an average of four line scans with a dwell time of 1.67 μs. BP filters 465-505+LP525 and beam splitters MBS 488/495 were used. Voxel dimensions were 0.068×0.068×0.3 μm. Z-stack consisted of 29 sections in total.

### Processing for 3D-EM

Unless otherwise stated, all EM reagents were purchased from TAAB Laboratories Equipment Ltd. Samples were incubated in reduced osmium (OsO_4_) [2% osmium tetroxide in de-ionized H_2_O (dH_2_O): all water used throughout was 18.2 MOhm/cm], 1.5% potassium ferrocyanide in 0.1 M sodium cacodylate buffer for 1 h at room temperature (RT). Samples were washed 5×3 min in dH_2_O, before incubation in 0.1% tannic acid (a mordant) in dH_2_O for 20 min at RT. An alternative to tannic acid is thiocarbohydrazide (1%), which is also capable of staining membranes (Seligman et al., 1966). After 5×3 min washes in dH_2_O, samples were osmium stained for 40 min using 2% OsO_4_ in dH_2_O at RT, then washed 3×5 min in dH_2_O and incubated with 1% uranyl acetate (UA) in dH_2_O overnight at 4°C. The next day, samples were washed 5×3 min in dH_2_O before incubation in Walton's Lead Aspartate (0.02 M lead nitrate, 0.03 M in aspartic acid in dH_2_O, adjusted to pH 5.5) for 30 min at 60°C (Walton, 1979). Dehydration of samples used a series of graded ethanol washes (30%, 50%, 70%, 90% in dH_2_O), followed by two washes in 100% ethanol. Samples were then infiltrated with TAAB Hard Premix resin washes using resin:ethanol mixes at ratios of 1:3, 1:2, 1:1, 2:1, 30 min incubation for each infiltration at RT. They were then covered in 100% resin and cured for 48 h at 60°C.

### Preparation of block and acquisition of EM data

Cured resin was separated from the gridded dish by trimming away the excess plastic and carefully sliding a razor between the dish and the resin. Excess resin was removed using a junior hacksaw and scalpel before the block was mounted onto a cryo pin, cell side up, using a conductive silver epoxy compound. Targeted trimming was performed using a UC6 ultra-microtome (Leica) as previously described (Booth et al., 2013). Samples were painted with Electrodag silver paint (avoiding the block face) and then coated with 10 nm AuPd using a Q150T sputter coater (Quorum Technologies). The sample was inserted into the Gatan 3View sample holder and adjusted so the block face would be central in the microtome and parallel with the knife-edge. After loading into the Gatan 3View microtome, which is mounted in a Quanta FEG250 ESEM (FEI), the sample height was raised manually using the dissecting microscope until the block face was close to the height of the knife. The final approach of the block face to the knife was achieved using the automatic approach on Digital Micrograph (Gatan) at 200 nm. Progressive low magnification ‘survey’ images (at 600× magnification; 200 nm sections) were first acquired from the block with continued reference to optical images. Once a suitable landmark or ROI had been identified, appropriate section thickness and acquisition settings were established (kV 2.3, image size, 3180×6954; dwell time, 15μs; magnification 1972×; final pixel size, 13.8 nm; section thickness, 80 nm).

EM datasets were batch-converted into tiff files in preparation for modelling in Amira™ (FEI). CLEM registration was performed using primary (EM) and secondary (LM) overlays with the ‘multiplanar’ tool, to confirm successful correlation of images.

### Shift analysis (Fiji)

To account for differences in section thickness (between LM and EM datasets), projections were generated from any sections that contained axons. The optical stack projection consisted of 9×300 nm sections (2.7 µm in total). The EM projection consisted of 33×80 nm sections (2.64 µm in total). A grid of concentric rings (‘concentric circles’, 6.5 µm spacing) was placed over both projections, centred on a clear landmark observable in both projections. Next, the position of structures/objects clearly identifiable in both projections (myelin, Fluoromyelin Green; nuclei, DAPI) were marked. The resulting coordinates were mapped onto a 2D-scatter plot and the ‘shift’ distance for X and Y, between LM and EM, was measured for each point. Using the same dataset both the distance of data points from origin (the landmark used to centre the shift analysis), and directional location (presented as the sin^−1^ angle of origin) of data points were also measured.

### 3D volume rendering of EM data using Amira™

Semi-automated segmentation of EM data was performed using the blow tool, allowing parameters such as volume and surface area to be measured. The blow tool acts as a suitable compromise between automation and accuracy. For example, all segmentation could be done totally manually, however this would be time consuming and subject to bias. Using tools that are totally automated removes all bias but is often poor at sufficiently segmenting fine structural details. The blow tool is a ‘drag-and-drop’ tool that uses a polygon expansion based on contrast gradient. In the case of myelin (which is very dark), this tool works very well in most cases. A more comprehensive guide is provided below (also refer to Fig. S1G-numbers refer to the numbered arrows in the figure).

EM stacks were imported into Amira™ and the appropriate voxel dimensions inputted when prompted.

1. Select the ‘Segmentation’ tab (arrow 1) to begin annotating the EM data (Fig. 4Ci).
2. Create a ‘new material’ and label it appropriately (e.g. Myelin) and choose a render colour.
3. Choose an optimal brightness/contrast using the virtual slider under ‘Display Control’. If comparative analyses of datasets are expected, make a note of the brightness/contrast values to provide consistency between datasets.
4. Select the blow tool. [Note: other segmentation tools (lasso, magic wand and threshold) can be used with varying degrees of success and levels of manual input. Our preference for segmenting myelin is the semi-automated blow tool. Once appropriate criteria have been established (for example adjustment of brightness/contrast and selection of structures of interest) we feel that the blow tool provides suitably unbiased (machine led) segmentation that is largely automated and relatively quick.]
5. Drag the blow tool across regions of myelin.
6. A preliminary boundary will be marked red on both the 2D orthoslice and, if using ‘Two Viewers’ (recommended), the model viewer.
7. Once satisfied with the annotations click ‘Add’. Segmented selections will now fix and appear green (colour selected earlier).
8. Continue segmentations through all orthoslices until the desired 3D model has been curated.
9. Return to ‘Project’ screen.
10. A new green node will have been created with a ‘labels’ suffix, plus any additional materials that have been created, such as ‘Myelin’. To generate a surface render, click on the small grey arrow and select the ‘Generate Surface’ option.
11. A new pink node, ‘Generate Surface’ will appear. Click on this tab, followed by ‘Apply’ located at the bottom of the Properties window.
12. To render the surface of segmented structures of interest click on the new tab with a ‘surf’ suffix and select the ‘Surface View’ option. A 3D surface view will be generated.
13. Select the ‘Four Viewers’ tab to view the model from multiple angles.

## Supplementary Material

Supplementary information
