# Supplementary material for: SuperCLEM: an accessible correlative light and electron microscopy approach for investigation of neurons and glia in vitro
Source: Biol Open. 2019 May 20;8(5):bio042085. doi: 10.1242/bio.042085 (PMC6550067; doi:10.1242/bio.042085)
Supplement: Supplementary information [file biolopen-8-042085-s1.pdf]

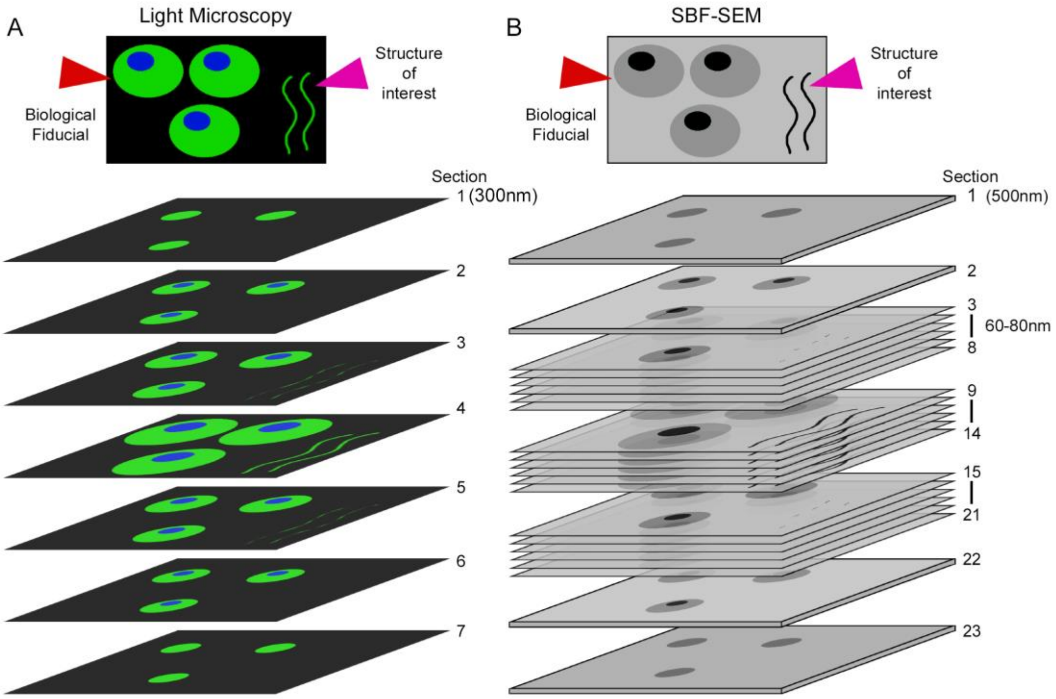

Fig. S1. Using biological fiducials for LM and SBF-SEM correlation.
Schematic to show the importance of identifying biological fiducials to act as reference points between LM (A) and SBF-SEM (B) data. In this example cell soma (red arrows) acted as “landmarks” as these large structures occupy many more Z sections than the target structures (pink arrows).

Biology Open • Supplementary information

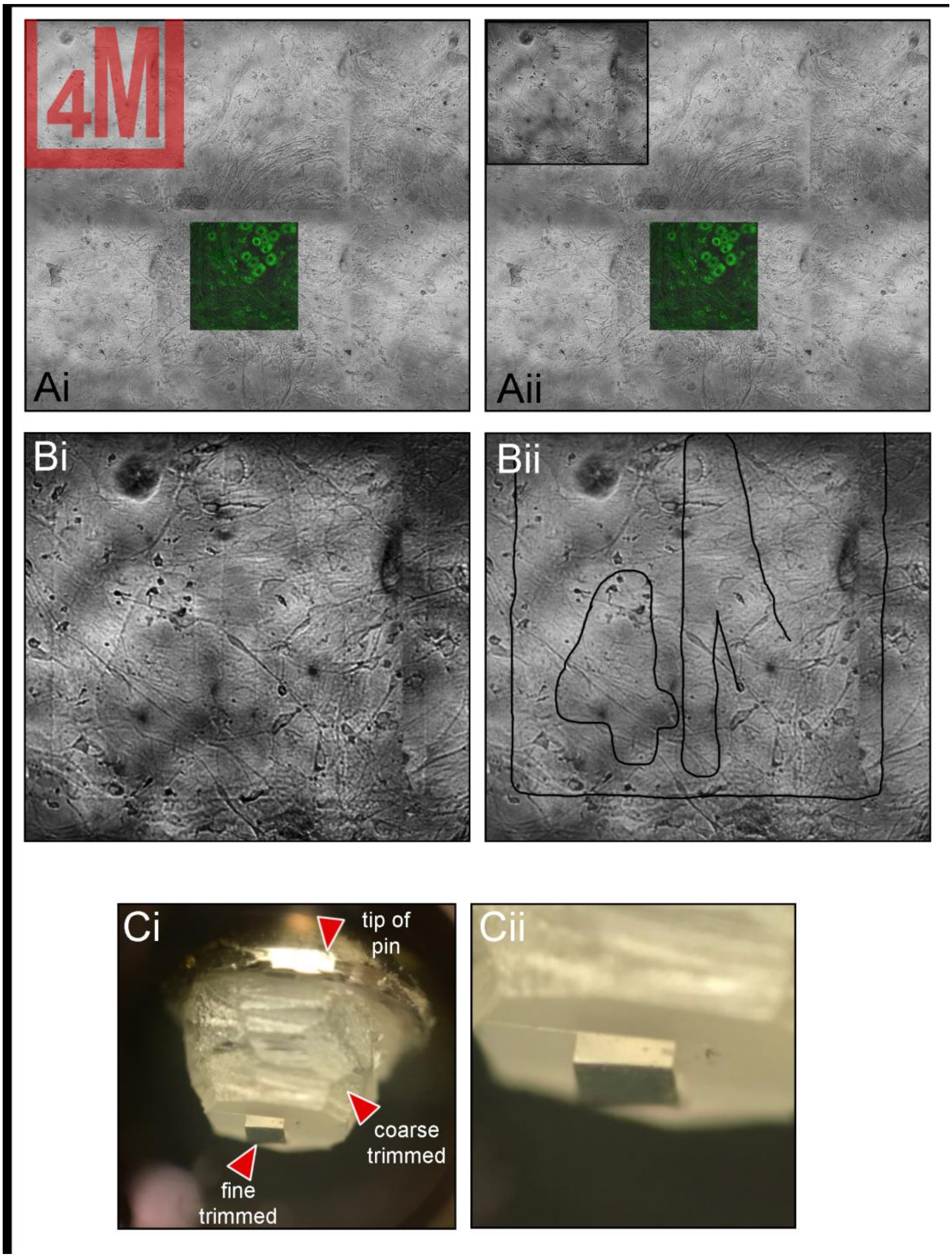

Fig. S2. Using tile-scans to reveal distal coordinates.

A) Dense beds of DRG axons masked the coordinates immediately beneath them. However, a tile-scan allowed the next nearest coordinate to be identified. In this sample 4M was the next nearest coordinate. B) Zoom of the area marked in Aii, either alone (Bi), or marked to highlight the letter/number (Bii). C) Additional versions of the block, labelled (Ci) and a zoom (Cii), as shown in Fig. 1Fiv.

Biology Open • Supplementary information

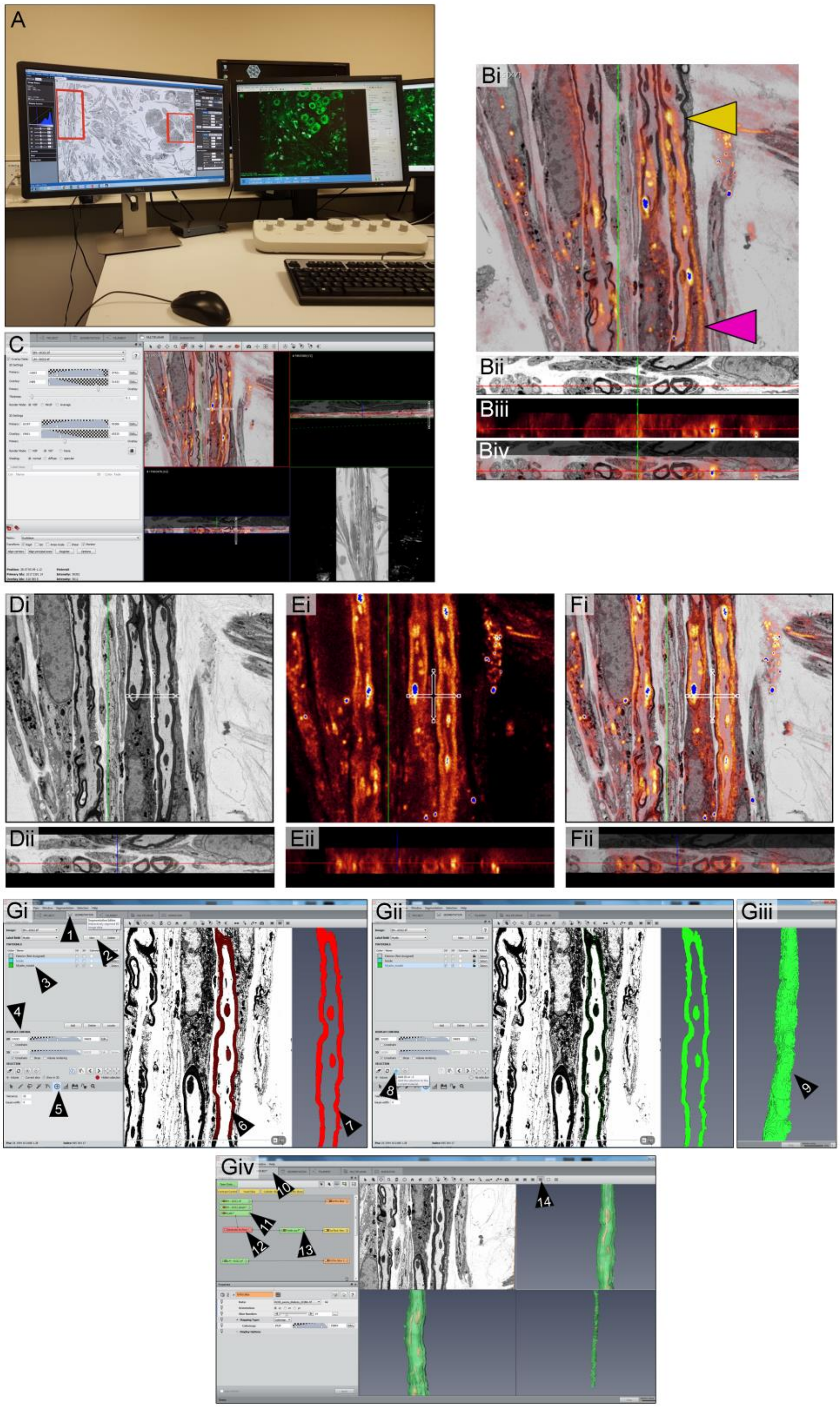

Fig. S3. *SuperCLEM* post-acquisition analysis.

A) CLEM workstation combining the use of at least two monitors is shown. The independent monitors allow "real-time" EM data to be viewed and compared with

Biology Open • Supplementary information

previously acquired LM data-stacks. This set-up is helpful for identifying "land-marks", allowing the position of ROI in the LM data to be re-visited by EM. Bi) Representative overlay of LM and EM sections showing imperfect correlation, in both the Z axis (yellow arrow – structures present in the LM data but not yet present in the EM data) and X/Y axis (pink arrow). Bii-iv) Orthogonal views of the overlaid images in Bi. C) Interface of the Amira™ "multiplanar" tool, allowing image stacks to be digitally re-sectioned to test for better image registration. D-F) Re-alignment of (D) EM and (E) LM data, achieving improved image registration (F), shown in both longitudinal (upper panels) and orthogonal (lower panels) sections. G) Guidance for rendering of EM data using Amira™. Please refer to methods section for step-by-step details.

Biology Open • Supplementary information

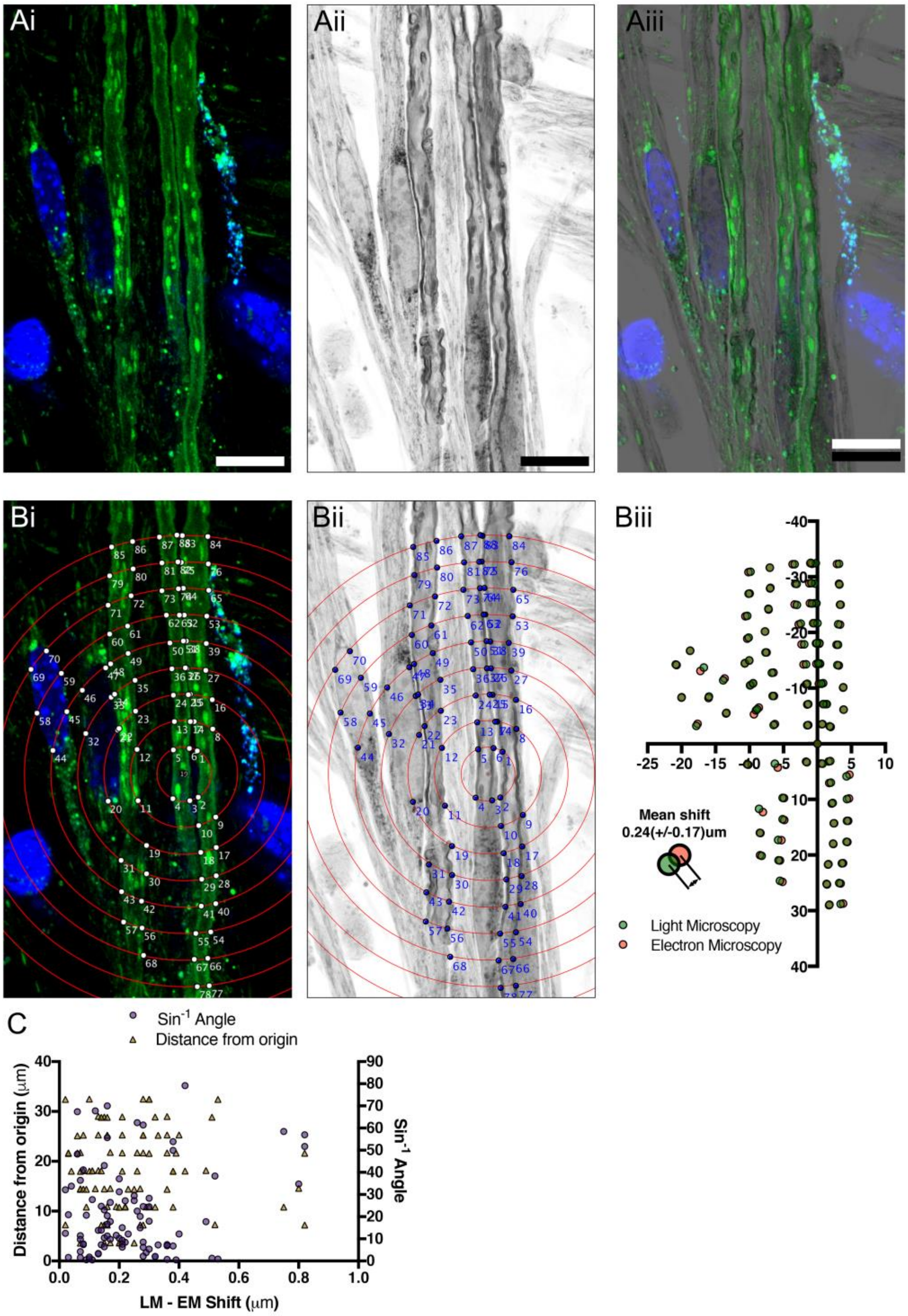

Fig. S4. Estimating the degree of correlation between LM and SBF-SEM data. A) Both LM (Ai) and SBF-SEM (Aii) sections that contained myelinated axons were projected into a single image. Aiii) Overlay of the LM and EM projections. Concentric rings were placed over the LM (Bi) and EM (Bii) projections and structures, clearly identifiable in both datasets, were marked. Biii) Scatterplot of the coordinates marked in Bi and Bii. C) Scatter plot of LM-EM shift, calculated in Biii (x axis) plotted against distance from origin (the origin in Biii) or angle (from Biii).

Biology Open • Supplementary information

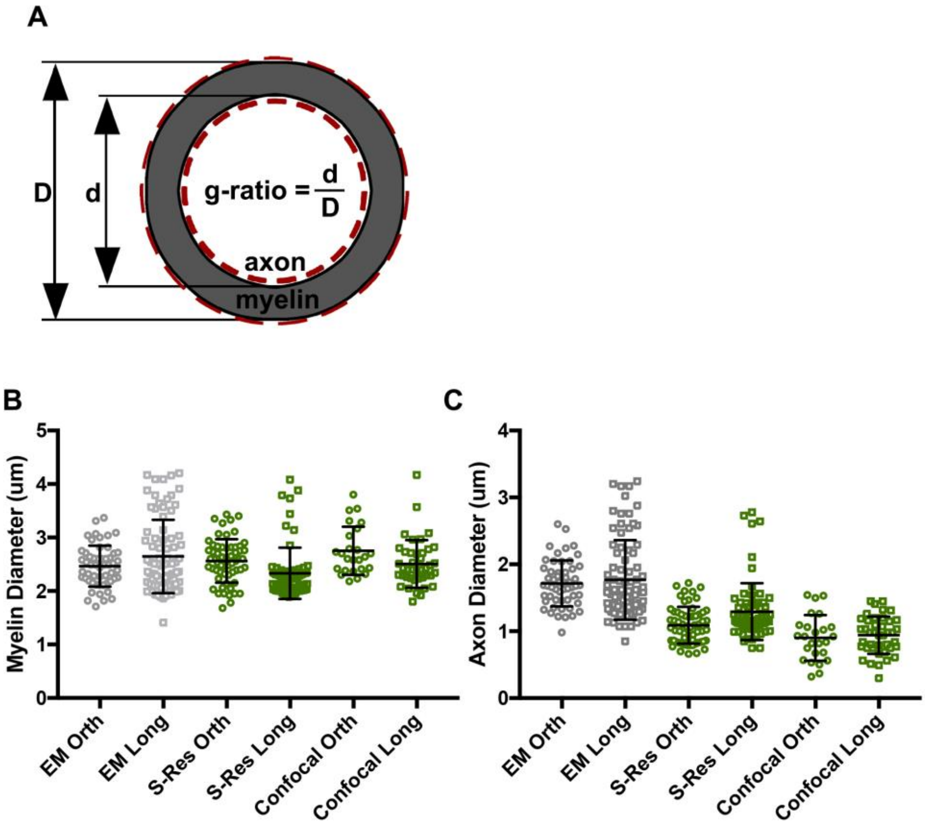

Fig. S5. Diameter measurements of myelin and axon.

A) Schematic showing the most commonly used approach to measure g-ratio – diameter of axon (d) divided by diameter of fibre (D). B, C) Raw diameter data used to estimate g-ratios in fig. 4 D. Pixel density scans (examples shown in fig.4 B and C) were acquired from identical regions in both longitudinal and orthogonal orientations. Line scans were used to measure fibre/myelin diameter (B) and axon diameter (C). Each point represents one line-scan measurement. N=3 axons, N=100 line scans. Note that due to excessive 'noise' created by out of focused light, some measurements could not be obtained from matching regions found in confocal images (N=3 axons, N=45 line-scans).

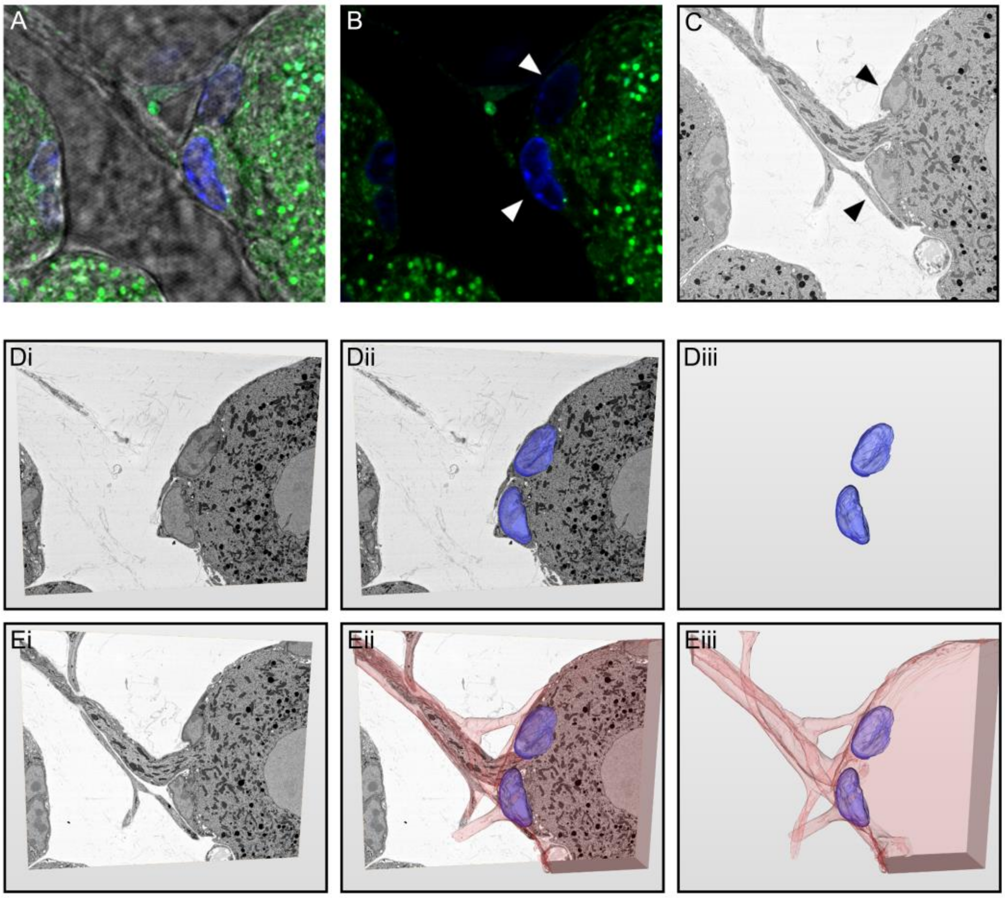

Fig. S6. SuperCLEM of an axon initial segment (AIS).

Other structures studied using SuperCLEM include an AIS. A) DAPI, Fluoromyelin Green and transmitted light. B) DAPI and Fluoromyelin Green. White arrows point to the nuclei of two satellite cells that envelope the sensory neuron. C) SBF-SEM orthoslice of an orthoslice matching the optical sections in A and B. Black arrows point to the same nuclei marked in B. D and E) Amira reconstruction of the AIS. The nuclei of satellite glial cells and the surface of the soma/AIS we rendered (in blue and red, respectively). D shows section 26 of 95. E shows section 67 of 95. Orthoslices alone (Di,Ei) orthoslices traversing the renders (Dii, Eii) and renders alone (Diii, Eiii) are shown.

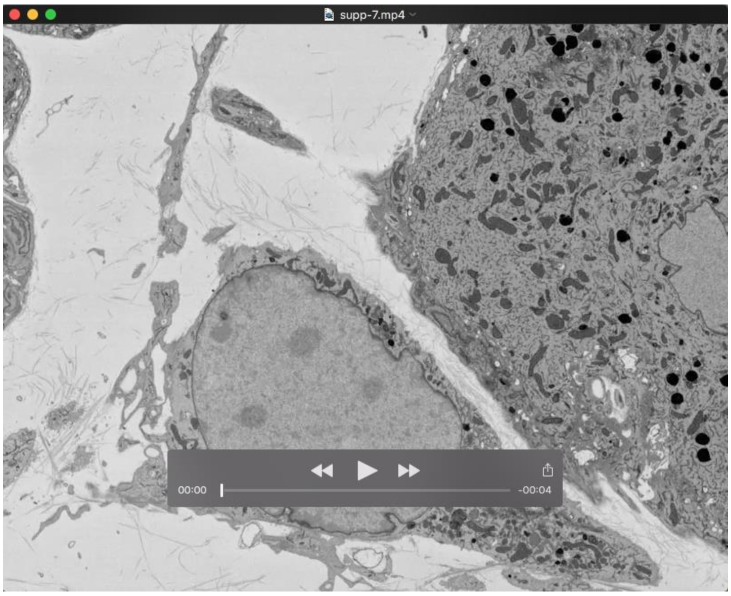

Movie 1. Digitally created model of a myelinated axon. Animation shows 3D-EM render of an in vitro myelinated axon analysed by *SuperCLEM* (black and white orthoslice). Digital models were segmented including, a Schwann cell nuclei (blue), an axon (yellow), myelin (green) and some invaginating regions of myelin (red).

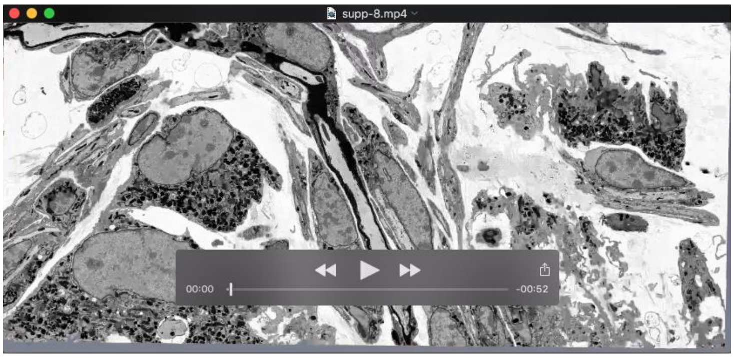

Movie 2. Movie of an axon initial segment (AIS). Video shows SBF-SEM stack of the AIS, traversing the Z plane.
